# The burden of mental health in lymphatic filariasis

**DOI:** 10.1186/s40249-015-0068-7

**Published:** 2015-07-30

**Authors:** Thanh G.N. Ton, Charles Mackenzie, David H. Molyneux

**Affiliations:** Precision Health Economics, 555 12 th Avenue, Suite 1650, Oakland, CA 94607 USA; Filariasis Programmes Support Unit, Department of Parasitology, Liverpool School of Tropical Medicine, Pembroke Place, Liverpool, L3 5QA UK; LSTM NTDs, Department of Parasitology, Liverpool School of Tropical Medicine, Pembroke Place, Liverpool, L3 5QA UK

**Keywords:** Lymphatic filariasis, Mental health, Global burden of disease, DALYS, Filariasis morbidity, Caregivers

## Abstract

**Background:**

Neglected Tropical Diseases (NTDs) afflict around one billion individuals in the poorest parts of the world with many more at risk. Lymphatic filariasis is one of the most prevalent of the infections and causes significant morbidity in those who suffer the clinical conditions, particularly lymphedema and hydrocele. Depressive illness has been recognised as a prevalent disability in those with the disease because of the stigmatising nature of the condition. No estimates of the burden of depressive illness of any neglected tropical disease have been undertaken to date despite the recognition that such diseases have major consequences for mental health not only for patients but also their caregivers.

**Methods:**

We developed a mathematical model to calculate the burden of Disability- Adjusted Life Years (DALY) attributable to depressive illness in lymphatic filariasis and that of their caregivers using standard methods for calculating DALYs. Estimates of numbers with clinical disease was based on published estimates in 2012 and the numbers with depressive illness from the available literature.

**Results:**

We calculated that the burden of depressive illness in filariasis patients was 5.09 million disability-adjusted life years (DALYs) and 229,537 DALYs attributable to their caregivers. These figures are around twice that of 2.78 million DALYs attributed to filariasis by the Global Burden of Disease study of 2010.

**Conclusions:**

Lymphatic filariasis and other neglected tropical diseases, notably Buruli Ulcer, cutaneous leishmaniasis, leprosy, yaws, onchocerciasis and trachoma cause significant co morbidity associated with mental illness in patients. Studies to assess the prevalence of the burden of this co-morbidity should be incorporated into any future assessment of the Global Burden of neglected tropical diseases. The prevalence of depressive illness in caregivers who support those who suffer from these conditions is required. Such assessments are critical for neglected tropical diseases which have such a huge global prevalence and thus will contribute a significant burden of co-morbidity attributable to mental illness.

**Electronic supplementary material:**

The online version of this article (doi:10.1186/s40249-015-0068-7) contains supplementary material, which is available to authorized users.

## Multilingual abstracts

Please see Additional file 1 for translations of the abstract into the six official working languages of the United Nations.

## Background

The impact of mental illness on the sufferers of Neglected Tropical Diseases (NTDs) appears to have been completely disregarded as a cause of morbidity and was not quantified in recent estimates in the Global Burden of Disease (GBD) study [1, 2]. Estimates of burden of NTDs have focused on the pathology and the contribution of disease sequelae to morbidity and premature mortality in the calculation of disability-adjusted life years (DALYs) [1, 2]. However, we have recently highlighted the extent of mental illness on sufferers of NTDs, which cause chronic sequelae and long-term often stigmatising conditions as a consequence of irreversible pathology [3]. The characteristics of chronic NTDs include the inability to work of both patients and caregivers; a change to less productive employment resulting in reduced or lack of income; a burden of debt through catastrophic and often inappropriate health expenditure; an inability to pay for schooling thereby impacting on the life time consequences for children; and a reduced societal role. Associated stigma often prevents patients from playing a full role in society or from leading a fulfilling emotional life, often with reduced marital prospects impacting patients and families with potential lifelong continuity of care from failure of women to marry with repercussions not only for the patient but also the family [3–5].

The latest GBD study [1] attributes mental and behavioural disorders as 7.4 % in 2010 of the total GBD of which 2.5 % was due to major depressive disorders and 1.1 % to anxiety disorders. In these calculations it is unclear if any burden is attributable to the mental health of those suffering from an NTD has been included. Hotez et al. [2] reviewed the Burden of NTDs [1] but did not include in any of the estimates the burden of mental health associated with NTDs. The GBD of 2010 [1] attributes to NTDs 1 % of the total global DALYs (excluding malaria) but the burden of NTDs varies regionally by a factor of 961 reflecting the relative importance of these diseases in the impoverished tropics where NTDs are amongst the commonest infections and where transmission remains intense. The GBD states “as most of these diseases cause limited mortality” a statement that we dispute, the NTDs highlight why quantification of disability from diseases is important. The follow up paper on NTDs [2] and the GBD study point out the apparent shift in global burden of disease from communicable to non- communicable diseases - a conclusion that impacts global health policy recommendations. However, the role of NTDs in causing cancers (schistosomiasis, food borne trematodes), cardiovascular and liver disease, neurological sequelae (human African trypanosomiasis, neurocysticercosis) and injuries (rabies and snake bite), are well recognised [6]. The impact on mental health of these outcomes of NTD infection has been ignored or disregarded in burden estimates [1, 2]. Furthermore, an unstudied dimension of NTDs, which has significant additional burden on poor communities, is that of the problems faced by caregivers who are committed to the long-term care of the disabled and who also suffer from the problems associated with both the direct and indirect costs of care [3]. A search of the literature has failed to find any study that examines the mental health of the caregivers of those suffering the chronic pathology of NTDs [3]. Studies in India on the burden of depression in caregivers of totally blind patients recorded a prevalence of depression of 48 % [7]. In the case of those with visual impairment, studies on the rates of depressive illness and diminished quality of life have been conducted, but until the recent study in India, a quantitative evaluation burden and depression in caregivers does not appear to have been undertaken in a developing world setting. There is a body of literature evaluating the problems faced by caregivers of patients with HIV/AIDs [8] and geriatric conditions [9, 10] and a number of well-defined instruments and methodologies for evaluating the care burden exist [9, 10]. The recent publication by World Health Organization (WHO) [8] on the “Lymphatic Filariasis: Managing Morbidity and Preventing Disability” states that “The psychological effects of lymphatic filariasis have been poorly recognized” and suggests a set of activities for psychological support for patients and for socioeconomic rehabilitation [11]. However, within the context of resource poor rural settings, there is unlikely to be either the specialist skills or resources to implement such support given the almost complete dearth of psychological or psychiatric expertise in such settings particularly in sub-Saharan Africa.

Studies on filariasis patients reports prevalence of depression or feelings of inferiority as high as 97 % in India [12]; in Togo, [13] 70 % of sufferers ranked high on the Duke Depression scale; in Haiti, 37 % were at risk of depression [14] although only 8 % prevalence was recorded in Sri Lanka [15]. Recent studies in Ethiopia [16] on podoconiosis have shown that patients suffering from the clinically similar lymphedema (notwithstanding the different aetiology) documented a higher burden of mental distress compared with healthy controls. The term mental distress in this study was defined as any form of emotional suffering due to podoconiosis and was deemed a more appropriate measure rather than a specific psychiatric diagnosis within the social and cultural setting of the study. In this paper, we present an estimate of the Global Burden of Mental illness associated with lymphatic filariasis based on the published figures of depressive illness from the studies quoted above [12–15], and include the estimates of depression, which can be attributed to caregivers based on estimates of the need for care by chronically disabled patients with filariasis.

## Methods

We abstracted estimates from the literature on lymphatic filariasis to populate our models. We used the WHO’s framework of burden of disease in terms of DALYs to estimate the mental health burden of lymphatic filariasis among patients and their caregivers. We calculated DALYs as the sum of Years of Life Lost (YLL) and Years Lost due to Disablity (YLD). YLD, is in turn, the product of the prevalence of the condition and the disability weights. Conceptually, the burden of depression attributed to filariasis represents a proportion of the total burden of filariasis, and is the sum of YLL and YLD for depression attributed specifically to filariasis. We abstracted estimates from the literature [17, 18] to populate our disease burden model, and used disability weights for mild, moderate and severe depression from the GBD [17].

## Results

The most recent estimate in 2012, which recognises the impact of the Global Programme to eliminate lymphatic filariasis, suggests 67.88 million people are infected with lympathic filariasis, 36.11 million with clinical illness, 19.43 million with genital hydrocele, and 16.68 million with lymphedema [19]. This is a decline from the previously quoted figures of WHO when the Global Programme to Eliminate the diseases began in 2000 when 119 million were estimated to be infected in 1996 with 43 million showing clinical symptoms (hydrocele and lymphedema). The DALYs attributable to lymphatic filariasis in the GBD study [1] were 2.78 million, based on a disability weight of 0.11 and 36 million cases (95 % confidence internal: 34–39 million) in 2010. In our studies to calculate the burden of depressive illness, we used disability weights of 0.159 for mild depression, 0.406 for moderate depression, and 0.655 for severe depression [17, 18]. Using a figure of 50 % that those with clinical illness suffer severely enough to cause depression, the prevalence of depression among patients with lymphatic filariasis was estimated at 18.1 million. This was based on the four studies of the mental illness in filariasis patients in the literature where the prevalence of depressive illness was 97 % in South India; 70 % in Togo; 37 % in Haiti and 8.5 % in Sri Lanka [12–15]. We used a conservative distribution estimate of ten million, seven million and one million for mild, moderate and severe depression, respectively. The studies from the above countries did not provide detailed breakdowns for the level of depression reported hence we chose a conservative set of estimates with only one million out of the 18 million sufferes of depression being regarded as having severe depression the majority only suffering mild depression. Clearly a higher figure of severe depression given the higher DW would result in a much higher estimate of burden. We estimated YLD to be 1.59 million, 2.84 million and 655,000 for these levels of depression severity, totalling 5.09 million DALYs. Because mortality directly attributable to lymphatic filariasis is rare although suicidal ideations have been reported, and because the GBD classified no deaths from depression in 2010, we assumed that YLL is close to zero. As a result, the total DALYs estimated from depression attributed to lymphatic filariasis is estimated at 5.09 million (Appendix A).

We then estimated mental burden of lymphatic filariasis among caregivers using similar assumptions (Appendix B). We further assumed that among the 36.1 million with clinical symptoms, five million will suffer severely enough to require a caregiver. Further assuming that one caregiver exists for each patient with severe symptoms, we estimated the total number of caregivers as five million which could be an underestimate. In a study of caregivers of blind individuals, 48 % had depression. To provide a conservative estimate, we assumed 25 % of caregivers of patients with lymphatic filariasis to have depression. We further assumed that caregivers with severe depression will select themselves out of the role of caregiving due to the disability associated with severe depression. Therefore, we assumed caregivers will have only mild or moderate depression. With a distribution of 90 % with mild depression and 10 % with moderate depression, we estimated the YLD as 178,887 and 50,750, for a total of 229,537. Because the GBD does not classify any deaths as attributable to depression, we approximated YLL as zero. As a result, the burden of depression among caregivers of patients with lymphatic filariasis is approximately 229,537 DALYs.

## Discussion

This paper is the first to attempt to quantify the burden of mental illness caused by an NTD, and indeed with one of the most prevalent of these diseases -lymphatic filariasis where, despite progress towards elimination [19, 20] with at least 4.9 billion treatments already given to around one billion people, millions with clinical disease remain [20]. We consider that the co-morbidity associated with mental illness of NTDs [3] is a seriously underestimated dimension of the global disease burden of many NTDs. We also recognize that there are limitations in the study given the paucity of data available to produce more accurate estimates of depressive illness for LF as well as other NTDs. However, given that LF is endemic in some 70 plus countries and notwithstanding the success of the Global Programme to eliminate the disease we believe that using the available data from what WHO considers as one of the most disabling infections represents an important starting point to encourage the need for more detailed studies not only in LF but also other NTDs. We also justify the inclusion of data on caregivers as an example of the total neglect of this aspect of co-morbidity as a result of NTDs. The GBD study of 2010 [1] and the recent evaluation of the NTD burden [2] did not address the mental health morbidity of NTDs. In addition, the mental health consequences of NTDs have not been considered in the overview of mental health and substance use disorders [21] in the GBD 2010 study, nor were NTDs considered in a major review of mental disorders in 2008 [22]. Given the numbers who suffer globally from the consequences of NTDs we consider this a major omission in any debate. The DALY burden of lymphatic filariasis was calculated as 2.78 million [1, 2]. We have used in our calculations the estimates of Ramaiah and Ottesen [19] of 2012 data, which recognise the impact of the Global LF Elimination Programme following 12 years of implementation of mass drug administration in 60 countries and where annual treatments in 2013 was 410 million reported by countries [20]. The figures for the numbers of individuals treated and the impact of the GPELF are provided in [19] but there are only a small number of papers in the published literature that provided prevalence data on mental health amongst those with clinical filariasis; it was thus necessary to make some conservative assumptions based on the wide range of prevalence of mental illness reported [12–15]. We recognize that the four studies which we used to inform our calculations from diverse settings cannot be considered as necessarily representative. However they are the only studies available on which to base calculations. Given that we have estimated that 50 % of those suffering from filariasis had an overall prevalence of depression we consider that figure a conservative estimate. This figure, however, could be much higher as for example the study in India recorded 97 % depressive illness in patients. Given that the numbers of LF cases in India is the highest in the world then our estimates could be considered significantly higher than we calculated. In addition, following an earlier study, we have estimated a further burden of associated depression based on the role that caregivers play in patient support. We also consider that the limitations of the estimates of depression in caregivers must be recognized. However, we include these results as patient dependency on care when severly disabled has not been recognized in any study of NTDs. Again we have been cautious about the parameters we have chosen to make these calculations but emphasise that further studies are needed not only in lymphatic filariasis but also in other NTDs where care of sufferers is necessary with consequent implications for families and communities.

Our estimate of 5.09 million DALYs of the burden associated with our estimates of the mental health burden alone of lymphatic filariasis contrasts with the total burden of 2.78 million attributed in the 2010 GBD study [1] and the NTD reconciliation [2] of that study for NTD diseases. We suggest that the burden of mental health, using conservative estimates of the prevalence of mental illness in LF, and the disability weights attributable to depressive illness used in the GBD study suggest that the burden of total LF is some three times the estimate of 2.78 million DALYs and that mental health contributes to twice the burden of morbidity compared with that published by GBD 2010 for lymphatic filariasis [1, 2]. It is possible that GBD reached this estimate by including a prevalence number of 25 million – almost half that of what we used. The GBD 2010 project obtained estimates of prevalence using a Bayesian modeling software. This method may result in prevalence estimate that differs from those reported by the WHO.

We recognize there may be limitations in our estimates as we used deterministic mathematical models that do not incorporate stochastic methods to account for uncertainty and we made assumptions in our calculations based on estimated prevalence and published literature combined with several additional assumptions on the distribution of severity of depression. However, we consider that despite this the burden of mental illness, which can be attributed to filariasis, has been grossly underestimated and that using this approach to calculating the burden of mental illness should be applied to other stigmatising NTD diseases [3] where there are known to be psychiatric sequelae for patients and their caregivers.

Having identified this often unrecognised area in defining the true medical burden of an NTD like filariasis, it is important to further comment on the need to establish appropriate methods to assess mental health at the field level in these programmes. A number of tools are available, such as the Mental Health Questionnaire (MHI-5), the General Health Questionnaire (GHQ-12), the Implicit Association test (IAT) for stigma assessment and the Four Dimensional Symptom Questionnaire, for distress and mental health in working populations. Which tool is most appropriate needs investigation. It should also be recognised that in NTDs there are two levels of causation for a patient’s depression, that caused by the stigma associated with the actual physical changes caused by the disease (e.g. lymphoedema or hydrocele in LF), and on a secondary level the further isolation and depression resulting from being stigmatised as being mentally affected by that primary LF –induced mental illness. This underscores the complexnature of the mental changes seen in NTD patients that need more complicated, or at least special, investigation methods and assessments. Focusing on appropriate ways of assessing the mental health aspects of NTDs, as emphasised in this discussion, will undoubtedly bring us close to truly understanding the importance of this aspect of morbidity management of this important group of global diseases.

## Conclusions

The burden of mental illness associated with neglected tropical diseases has not been calculated. We have used published literature of the prevalence of mental health in lymphatic filariasis patients to calculate the Disability Adjusted Life Years (DALY) burden based on the most recent estimates of global morbidity. We estimate that the burden of mental illness due to depressive illness is 5.09 million DALYS whilst the burden of mental illness in caregivers is 229,537 DALYS. These figures are approximately twice the figure estimated in the 2010 GBD study for lymphatic filariasis of 2.78 million DALYS. We consider that the burden of mental illness of other NTDs needs to be assessed, as to date, this crucial element of morbidity is not reported as a contributor to the global burden of disease and no studies have been undertaken on the burden of mental illness in caregivers.

## Appendix A

### Global burden of depression caused by filariasis among patients

A. Background information
  Filariasis assumptions [17, 19]
  - Number infected with lymphatic filariasis 67.88 million
  - Number with clinical illness 36.11 million
    - Genital hydrocele 19.43 million
    - Lymphodema 16.68 million
  - Estimated with symptoms of depressive illness 18.1 million
  - Of those with depression, 50 % had moderate or severe 9.0 million
  - GBD disability weight for filariasis 0.11
  - Years of life lived with symptoms 20 years
B. Objective
  Estimate the burden of depression caused by filariasis?
C. Overall DALY framework
  Burden of any disease is mathematically estimated as:
  1 $$ \mathrm{DALY} = \mathrm{Y}\mathrm{L}\mathrm{L} + \mathrm{Y}\mathrm{L}\mathrm{D} $$
  - where YLL = years lost to premature death caused by that disease, and
  - where YLD = years lived with disability caused by that disease
  In this situation, we are interested in the burden of a particular disease, depression, that is specifically attributed to a particular neglected tropical disease, filariasis.
  We modified this mathematical formula for burden of depression caused by filariasis such that the burden of depression would be conditioned on filariasis as a cause:
  2 $$ {\mathrm{DALY}}_{\left[\mathrm{depression} \sim \mathrm{filariasis}\right]} = {\mathrm{YLL}}_{\left[\mathrm{depression} \sim \mathrm{filariasis}\right]} + {\mathrm{YLD}}_{\left[\mathrm{depression} \sim \mathrm{filariasis}\right]} $$
  In other words, we estimated the burden of depression caused only by filariasis (DALY_[depression ~ filariasis]_). Conceptually, this quantity is a proportion of the overall DALY for filariasis:
  3 $$ {\mathrm{DALY}}_{\left[\mathrm{depression} \sim \mathrm{filariasis}\right]}=q*{\mathrm{DALY}}_{\left[\mathrm{filariasis}\right]} $$
D. Component of DALY: years lived with disability (YLD)
  We know that mathematically:
  4 $$ \mathrm{Y}\mathrm{L}\mathrm{D} = \mathrm{P}\ \mathrm{x}\ \mathrm{D}\mathrm{W} $$
  - where P = number of prevalent cases
  - where DW = disability weights
  Within our context of filariasis and depression:
  5 $$ {\mathrm{YLD}}_{\left[\mathrm{depression}\sim \mathrm{filariasis}\right]} = {\mathrm{P}}_{\left[\mathrm{depression}\sim \mathrm{filariasis}\right]}\mathrm{x}\ {\mathrm{DW}}_{\left[\mathrm{depression}\right]} $$
  - where = P_[depression~filariasis]_ = prevalence of depression among those with filariasis
  - where DW_[depression~filariasis]_ = disability weights of having depression
  - 50 % of those with filariasis suffer severely enough to cause depression. Therefore, P = 20 million
  - Eaton and colleagues used a weight of 0.35 for major depressive disorders in the 1996 GBD project.^22^ To provide a relative context of disability weights, the following are weights noted by Eaton and colleagues: quadriplegia is 0.90, for blindness is 0.62, for multiple sclerosis is 0.41, for deafness is 0.33, for rheumatoid arthritis is 0.21, and for watery diarrhea is 0.07.
  - In 2010, GBD estimated 3 separate disability weights for mild, moderate and severe depressive symptoms: 0.159, 0.406, 0.655.^17^
  Assuming the distribution of mild, moderate, and severe depression, we estimate the following components for DALYs:
  **Table 1:** ᅟ
  Parameter	Estimate for mild depression	Estimate for moderate depression	Estimate for severe depression	Sum
  P	10 million	7 million	1 million	18. million
  DW	0.159	0.406	0.655	n/a
  YLD (=PxDW)	1.59 million	2.84 million	655,000	5.09 million
E. Component of DALY: Years of Life Lost (YLL)
  The second component of DALYs is YLL:
  6 $$ \mathrm{Y}\mathrm{L}\mathrm{L} = \mathrm{N}\ \mathrm{x}\ \mathrm{L} $$
  - where N = number of deaths
  - where L = standard life expectancy at age of death
  Within the context of filariasis and depression, we modify the above formula accordingly:
  7 $$ {\mathrm{YLL}}_{\left[\mathrm{depression}\sim \mathrm{filariasis}\right]} = {\mathrm{N}}_{\left[\mathrm{depression}\sim \mathrm{filariasis}\right]}\mathrm{x}\ {\mathrm{L}}_{\left[\mathrm{depression}\sim \mathrm{filariasis}\right]} $$
  - Where N_[depression~filariasis]_ = number of deaths from depression caused by filariasis
  - Where L_[depression~filariasis]_ = life expectancy of depression caused by filariasis
  Conceptually, the number of deaths caused by depression attributed to filariasis, N_[depression~filariasis]_, will be a number of those who have depression caused by filariasis and who go on to die as a result. According to GBD, no deaths occurred as a result of depression in 2010. In addition, according to the WHO, although filariasis is one of the leading causes of disability, death from filariasis is rare.
  Therefore, we will assume N_[depression~filariasis]_ ~ 0
F. Calculating DALY from YLL + YDL

If put all of our estimates together, we will have:

**Table 2:** ᅟ

	YLL	YLD (million)	DALYs (million)
Disability from depression attributed to filariasis	0	5.09	5.09

In summary, we estimate that the burden of depression attributed to filariasis in 2010 is approximately 5.09 million.

## Appendix B

### Global burden of depression caused by filariasis among caregivers

A. Background Information
  Filariasis assumptions
  - clinical sx 36.1 million
  - prevalence of filariasis with very severe symptoms 5 million
  - number of caregivers of very severe filariasis (1:1) 5 million
  - proportion of caregivers who have depression 25 %
  - years life lived with filarisis 20 years
B. Objective
  Estimate the burden of depression caused by taking care of an individual with severe filariasis.
C. Overall DALY framework
  We know that the burden of any disease is mathematically estimated as:
  8 $$ \mathrm{DALY} = \mathrm{Y}\mathrm{L}\mathrm{L} + \mathrm{Y}\mathrm{L}\mathrm{D} $$
  - where YLL = years lost to premature death caused by that disease, and
  - where YLD = years lived with disability caused by that disease
  In this situation, we want to know about the burden on caregivers who take care of individuals with severe filariasis.
  Our mathematical formula for burden of depression caused by taking care of an individual with severe filariasis would have to be applied to caregivers, as opposed to patients. We do not expect that caregivers to be fundamentally different from other individuals in terms of experiencing disease burden; therefore, we apply the same formula for disease burden for caregivers:
  9 $$ {\mathrm{DALY}}_{\left[\mathrm{depression}\sim \mathrm{caring}\ \mathrm{f}\mathrm{o}\mathrm{r}\ \mathrm{f}\mathrm{ilariasis}\right]} = {\mathrm{YLL}}_{\left[\mathrm{depression}\sim \mathrm{caring}\ \mathrm{f}\mathrm{o}\mathrm{r}\ \mathrm{f}\mathrm{ilariasis}\right]} + {\mathrm{YLD}}_{\left[\mathrm{depression}\sim \mathrm{caring}\ \mathrm{f}\mathrm{o}\mathrm{r}\ \mathrm{f}\mathrm{ilariasis}\right]} $$
  In other words, we are estimating the burden of depression caused by caring for those with severe filariasis (DALY_[depression ~ caregiving for filariasis]_).
D. Component of DALY: Years Lived With Disability (YLD)
  Calculation of YLD follows the formula:
  10 $$ \mathrm{Y}\mathrm{L}\mathrm{D} = \mathrm{P}\ \mathrm{x}\ \mathrm{D}\mathrm{W} $$
  - where P = number of prevalent cases
  - where DW = disability weights
  (This formula, YLD = P x DW, was used in GBD 2010 rather than what was used previously in earlier versions of GBD methodology: YLD = I x DW x L).
  Conceptually, this quantity is a proportion of the overall DALY of depression caused by caring for all other conditions.
  11 $$ {\mathrm{YLD}}_{\left[\mathrm{depression}\sim \mathrm{caring}\ \mathrm{f}\mathrm{o}\mathrm{r}\ \mathrm{f}\mathrm{ilariasis}\right]} = {\mathrm{P}}_{\left[\mathrm{depression}\sim \mathrm{caring}\ \mathrm{f}\mathrm{o}\mathrm{r}\ \mathrm{f}\mathrm{ilariasis}\right]}\mathrm{x}\ {\mathrm{DW}}_{\left[\mathrm{depression}\right]} $$
  - where = P_[depression~caring for filariasis]_ = number of depression caused by caring for severe filariasis
  - where DW_[depression~caring for filariasis]_ = disability weights of depression
  We obtained parameters from the literature and the following assumptions:
  - For P, the prevalence of caregivers of individuals with filariasis is five million. We assumed that 25 % of these five million will have depression. This estimate is obtained from literature on prevalence of depression among caregivers for the blind (48 %), and have reduced this proportion to 25 % for lympathic filarisis to obtain a conservative estimate. As a result, P = 1,250,000 cases of caregivers for filariasis who have depression.
  - The burden of depression is given by disability weights: 0.35^3^
  Here is a summary of our estimates:
  **Table 3:** ᅟ
  Parameter	Estimate or Range	Comments
  P	1.25 million	25 % of 5 million caregivers assumed to have depression
  DW for depression	0.159, 0.406, 0.655	Obtained from Salomon et al.
  The calculation of YLD from P and DW with 1.25 million caregivers suffering from mild to moderate depression is described below. We assumed that caregivers with severe depression will select themselves out of the role of caregiving given the high disability associated with severe depression:
  **Table 4:** ᅟ
  Depression	Distribution		DW	YLD
  Mild	90 % of 1.25 mill	1,125,000	0.159	178,887
  Moderate	10 % of 1.25 mill	125,000	0.406	50,750
  Sum	100 %	1,250,000	n/a	229,637
E. Component of DALY: Years of Life Lost (YLL)
  The second component of DALYs is YLL and is calculated as:
  12 $$ \mathrm{Y}\mathrm{L}\mathrm{L} = \mathrm{N}\ \mathrm{x}\ \mathrm{L} $$
  - Where N = number of deaths
  - Where L = standard life expectancy at age of death
  Within the context of depression experienced by caregivers of individuals with severe filariasis, we modify the above formula accordingly:
  13 $$ {\mathrm{YLL}}_{\left[\mathrm{depression}\sim \mathrm{caring}\ \mathrm{f}\mathrm{o}\mathrm{r}\ \mathrm{f}\mathrm{ilariasis}\right]} = {\mathrm{N}}_{\left[\mathrm{depression}\sim \mathrm{caring}\ \mathrm{f}\mathrm{o}\mathrm{r}\ \mathrm{f}\mathrm{ilariasis}\right]}\mathrm{x}\ {\mathrm{L}}_{\left[\mathrm{depression}\sim \mathrm{caring}\ \mathrm{f}\mathrm{o}\mathrm{r}\ \mathrm{f}\mathrm{ilariasis}\right]} $$
  - Where N_[depression~caring for filariasis]_ = number of deaths of caregivers of filariasis from depression
  - Where L_[depression~caring for filariasis]_ = life expectancy of caregivers of filariasis with depression
  Conceptually, the number of deaths caused by depression attributed to filariasis, N_[depression~caring for filariasis]_, will be a number of those who have depression caused by caring for individuals with severe filariasis and who go on to die as a result. According to GBD, no deaths occurred as a result of depression in 2010. We also do not expect that caregiving is causes excess mortality. Therefore, we will assume N_[depression~caring for filariasis]_ ~ 0.
  14 $$ {\mathrm{YLL}}_{\left[\mathrm{depression}\sim \mathrm{caring}\ \mathrm{f}\mathrm{o}\mathrm{r}\ \mathrm{f}\mathrm{ilariasis}\right]} = {\mathrm{N}}_{\left[\mathrm{depression}\sim \mathrm{caring}\ \mathrm{f}\mathrm{o}\mathrm{r}\ \mathrm{f}\mathrm{ilariasis}\right]}\mathrm{x}\ {\mathrm{L}}_{\left[\mathrm{depression}\sim \mathrm{fcaring}\ \mathrm{f}\mathrm{o}\mathrm{r}\ \mathrm{f}\mathrm{ilariasis}\right]} $$
  YLL_[depression~caring for filariasis]_ =0
F. Calculating DALY from YLL + YDL

From our calculations above:

DALY_[depression~caring for filariasis]_ = YLL_[depression~caring for filariasis]_ + YLD_[depression~caring for filariasis]_

DALY_[depression~caring for filariasis]_ = 0 + 229,637

DALY_[depression~caring for filariasis]_ = 229,637

We estimate that the burden of depression on caregivers of those with clinically symptomatic filariasis is 229,637.

## Additional file

Additional file 1:
**Translation of the abstract into the six official working languages of the United Nations.**
